# Association between the proportion of globally sclerotic glomeruli and various morphologic variables and clinical data of IgA nephropathy patients

**DOI:** 10.12861/jrip.2012.10

**Published:** 2012-01-01

**Authors:** Hamid Nasri, Mohammad Reza Ardalan

**Affiliations:** ^1^Department of Nephrology, Division of Nephropathology, Isfahan University of Medical Sciences, Isfahan, Iran; ^2^Chronic Kidney Disease Research Center, Tabriz University of Medical Sciences, Tabriz, Iran

**Keywords:** Immunoglobulin A nephropathy, Oxford classification, Sclerotic glomeruli

## Abstract

**Introduction:** Immunoglobulin A nephropathy is the most common form of glomerular nephropathy among children and young adults.Objectives: To evaluate the possible correlation between the extent of sclerotic glomeruli and various morphologic variables and clinical data of IgA nephropathy (IgAN) patients, we conducted an observational study, on 136 IgAN patients’ biopsies.

**Patients and Methods:** Of the 136 patients, 94 (69.1%) were male. The mean age of the patients was 37.6 ± 13.4 years. The mean of serum creatinine was 1.94±3.7 mg/dl (median=1.2mg/dl), also mean of proteinuria was 1726±1247 mg/day (median=1500 mg/day). In this study of 14.9±3.7 glomeruli in biopsies, 2.5±3.2 (median=2) were globally sclerotic.

** Results:** In this study we found, significant positive correlation between proportion of globally sclerotic glomeruli and serum creatinine, amount of proteinuria, and also quantity of tubulointerstitial fibrosis. Also, in this study, the association of proportion of globally sclerotic glomeruli with M, E, S and T variables of Oxford classification was significantly positive.

**Conclusion:** We propose firstly that, sclerotic glomeruli reported routinely in the pathology reports of IgA nephropathy patients and secondly we suggest further investigations to possible inclusion of other morphologic variables like proportion of sclerotic glomeruli to Oxford classification of IgAN to widen the scope of this classification.

**Implication for health policy/practice/research/medical:**

In a study on 136 primary IgA nephropathy patients, Association of proportion of sclerotic glomeruli with proteinuria and serum creatinine and also with all four morphologic variables of Oxford classification was seen. We propose firstly that sclerotic glomeruli reported routinely in the pathology reports of IgA nephropathy patients and secondly we suggest fur¬ther investigations to possible inclusion of other morphologic variables, such as proportion of sclerotic glomeruli to Oxford classification of IgA nephropathy to widen the scope of this classification.

## Introduction

Immunoglobulin A nephropathy is the most common form of glomerular nephropathy among children and young adults (1,2). The disease is clinically and morphological heterogeneous and slowly progressive in patients, leading to renal insufficiency in 15–20% of patients over a decade and in 20–30% over 20 years (1,2). Numerous attempts have been made to describe factors that predict those patients expected to have poor outcomes (1-3). Various studies characterized, decrease renal function, proteinuria and the presence of tubulointerstitial fibrosis and glomerular sclerosis at presentation as predictors of loss of renal function (2-4). However, it is well not defined yet, whether the proportion of globally sclerotic glomeruli have significance with regard to recent Oxford classification for IgA nephropathy (IgAN) (1-4).

## Objectives

This study aimed to assess the possible association between the extent of global glomerular sclerosis and various clinical or morphologic lesion in a group of IgAN.

## Patients and Methods

### Patients

We performed this study based on the Oxford classification of IgAN after it was published in July 2009.

The pathologic diagnosis of IgAN requires the demonstration of IgA-dominant mesangial immune deposits through immunofluorescence (IF) microscopy while there was absence of C_1_q deposits. All renal biopsies were performed at private or university medical centers in Isfahan, Iran, from July 2009 to October 2012, these biopsy specimens were sent to a reference laboratory. None of the patients underwent treatment before the biopsy. Those biopsies that were less than 8 glomeruli were excluded from this study. Based on the questionnaires completed at the time of the patients’ admission for biopsies, laboratory data in the patients’ records and brief histories provided by referring physicians, we found no patient diagnosed as primary IgAN and no patient with a history of collagen vascular diseases, diabetes or liver cirrhosis.

### Histologic data

All kidney biopsies were prepared for light and direct IF microscopy. The tissues were fixed in 10% formalin for histologic sectioning. After an IF diagnosis of IgAN, we reviewed the histopathology glass slides to define the morphologic variables, which were classified according to the Oxford classification system (3,4). After selecting those biopsies that had prominent IgA deposition according to IF study and did not meet any exclusion criteria, we classified the glass slides according to Oxford classification.

### Definitions of morphologic variables of IgAN (Oxford classification)

We recorded the total number of glomeruli and the number of glomeruli with global sclerosis for each biopsy. We estimated the presence of mesangial hypercellularity (M), endocapillary proliferation (E**)**, segmental glomerulosclerosis (S) and the proportion of tubular atrophy and interstitial fibrosis, IF/TA (T), as defined in the Oxford-MEST classification (3,4).

### Clinical studies and laboratory data

We reviewed the patients’ medical records to obtain various demographic, clinical and laboratory data at the time of their biopsies and for follow-up. We gathered the following data at the time of biopsy: race, gender, age, serum creatinine and proteinuria (based on a 24-hour urine collection).

### Ethical issues

(1) The research followed the tenets of the Declaration of Helsinki; (2) informed consent was obtained; (3) the research was approved by the institutional review board.

### Statistical analysis

We determined the frequency, mean values and standard deviations .We used the Spearman’s correlation coefficient to check the correlations. Statistical analyses were done using SPSS 16 (SPSS Inc, Chicago, IL). P<0.05 was considered statistically significant.

## Results

In our observational study, we enrolled a total of 136 IgAN patient biopsies. Of the 136 patients, 94 (69.1%) were male. The mean age of the patients was 37.6±13.4 years. The mean of serum creatinine was 1.6±1.5 mg/dl (median=1.2 mg/dl). Also mean of proteinuria was 1726±1247 mg/day (median=1500 mg/day). In this study of 14.9±3.7 glomeruli in biopsies, 2.5±3.2 (median=2) were globally sclerotic. The morphologic variables of the Oxford-MEST classification are summarized in Table 1.

**Table 1:** Morphologic variables of Oxford classification of IgAN at the time of renal biopsy

**Histological findings (Oxford-MEST classification)**	
**Mesangial hypercellularity**	
M0/M1 (number)	46/90
**Endocapillary hypercellularity**	
E0/E1 (number)	90/46
**Segmental glomerulosclerosis**	
S0/S1 (number)	48/88
**Tubular atrophy/interstitial fibrosis(IF/TA)**	
T0/T1/T2(number)	67/46/23

In this study, there was significant positive correlation between proportion of globally sclerotic glomeruli and serum creatinine (p=0.302, p<0.001), amount of proteinuria (r=0.278, p=0.001) and also quantity of tubulointerstial fibrosis (r=0.631, p<0.001).

In this study, the association of proportion of globally sclerotic glomeruli and four pathology variables of Oxford classification were as follows:

Between proportion of globally sclerotic glomeruli and M variant (r=0.302, p<0.001), E variant (r=237, p=0.005), S variant (0.401, p<0.001) and T variant (r=0.631, p<0.0001).

## Discussion

In this study we found, significant positive correlation between proportion of globally sclerotic glomeruli and serum creatinine, amount of proteinuria, and also quantity of tubulointerstial fibrosis. Also, in this study, the association of proportion of globally sclerotic glomeruli with M, E, S and T variables of Oxford classification was significantly positive. Attempts were made to find which of the morphologic lesions has prognostic significance. Of them, the recent Oxford classification described four morphologic lesions which had prognostic significance independently (3,4). However, The Oxford study consisted a small number of patients (n=265 patients). Therefore, it is essential to conduct complementary studies using a larger number of patients or studies from different geographic areas and ethnicities to find the other important prognostic morphologic variables (3-5). To evaluate the possible association between the extent of sclerotic glomeruli and the level of serum creatinine and its clearance rate in patients with primary focal segmental glomerolusclerosis, Fakhrjou *et al*. conducted a study on 50 patients with biopsy-proven primary focal segmental glomerolusclerosis .They found that the proportion of global glomerulosclerosis was directly correlated with the serum level of creatinine and inversely with its clearance rate (6). Lv *et al*. studied 204 biopsy-diagnosed IgAN patients, who were followed for an average of 6.1 years (range, 4-15 years). They found, the renal survival rate following biopsy was 85.1% at the fifth year, and 77.1% at the 10th year. They also found, patients who were male, had hypertension, proteinuria of more than 1 g/day, renal impairment and a high histological grading were associated with poor prognosis. They concluded that patients with renal impairment, hypertension and advanced histological involvement had the highest risk for disease progression (7). Studies concerning the significance of global glomerulosclerosis in IgAN were quite scarce. Lemley *et al*. measured GFR annually for 4-5 years in 22 adult patients with diagnosed IgAN. They found, the rate of GFR change ranged from a loss of 41 ml/min/year to a gain of 8.6 ml/min/year. They found an optimum predictor set of five baseline variables: the percentage of glomeruli with global sclerosis, the fractional interstitial area, the serum creatinine, the average tuft volume of non-sclerotic glomeruli and the renal plasma flow (8).

## Conclusions

Our findings support the importance of sclerotic glomeruli due to its association with proteinuria and serum creatinine and also with all four morphologic variables of Oxford classification. We propose firstly that sclerotic glomeruli reported routinely in the pathology reports of IgA nephropathy patients and secondly we suggest further investigations to possible inclusion of other morphologic variables, such as proportion of sclerotic glomeruli to Oxford classification of IgAN to widen the scope of this classification.

## Conflict of interests

The authors declared no competing interests.

## Financial Disclosure

None.

## Authors' contribution

HN conducted the research. MRA and HN wrote the manuscript.

## Ethical considerations

Ethical issues (including plagiarism, misconduct, data fabrication, falsification, double publication or submission, redundancy) have been completely observed by the authors.

## References

[1] Julian BA, Waldo FB, Rifai A, Mesteckey J (1988). IgA nephropathy, the most common glomerulonephritis worldwide: a neglected disease in the United States?. Am J Med.

[2] Koyama A, Igarashi M, Kobayashi M (1997). Natural history and risk factors for immunoglobulin A nephropathy in JapanResearch Group on Progressive Renal Diseases. Am J Kidney Dis.

[3] Coppo R, Troyanov S, Camilla R, Hogg RJ, Cattran DC, Cook HT (2010). The Oxford IgA nephropathy clinicopathological classification is valid for children as well as adults. Kidney Int.

[4] Roberts IS, Cook HT, Troyanov S, Alpers CE, Amore A, Barratt J (2009). The Oxford classification of IgA nephropathy: pathology definitions, correlations, and reproducibility. Kidney Int.

[5] Bartosik LP, Lajoie G, Sugar L, Cattran DC (2001). Predicting progression in IgA nephropathy. Am J Kidney Dis.

[6] Fakhrjou A, Hashempour A, Shadravan S, Fouladi RF (2012). Association between the proportion of sclerotic glomeruli and serum creatinine in primary focal segmental glomerulosclerosis. Turk Patoloji Derg.

[7] Lv J, Zhang H, Zhou Y, Li G, Zou W, Wang H (2008). Natural history of immunoglobulin A nephropathy and predictive factors of prognosis: a long-term follow up of 204 cases in China. Nephrology (Carlton).

[8] Lemley KV, Lafayette RA, Derby G, Blouch KL, Anderson L, Efron B (2008). Prediction of early progression in recently diagnosed IgA nephropathy. Nephrol Dial Transplant.

